# Emerging Cellular Functions of Cytoplasmic PML

**DOI:** 10.3389/fonc.2013.00147

**Published:** 2013-06-06

**Authors:** Guoxiang Jin, Ying-Jan Wang, Hui-Kuan Lin

**Affiliations:** ^1^Department of Molecular and Cellular Oncology, The University of Texas MD Anderson Cancer Center, Houston, TX, USA; ^2^Department of Environmental and Occupational Health, Medical College, National Cheng Kung University, Tainan, Taiwan; ^3^The University of Texas Graduate School of Biomedical Sciences at Houston, Houston, TX, USA

**Keywords:** cytoplasmic PML, TGF-ß, apoptosis, antiviral response, tumorigenesis

## Abstract

The tumor suppressor promyelocytic leukemia protein (PML) is located primarily in the nucleus, where it is the scaffold component of the PML nuclear bodies (PML-NBs). PML-NBs regulate multiple cellular functions, such as apoptosis, senescence, DNA damage response, and resistance to viral infection. Despite its nuclear localization, a small portion of PML has been identified in the cytoplasm. The cytoplasmic PML (cPML) could be originally derived from the retention of exported nuclear PML (nPML). In addition, bona fide cPML isoforms devoid of nuclear localization signal (NLS) have also been identified. Recently, emerging evidence showed that cPML performs its specific cellular functions in tumorigenesis, glycolysis, antiviral responses, laminopothies, and cell cycle regulation. In this review, we will summarize the emerging roles of cPML in cellular functions.

## Introduction

The. promyelocytic leukemia protein (PML) was initially identified in the acute promyelocytic leukemia (APL), a disease largely associated with t(15, 17) chromosomal translocation, which results in the fusion protein of PML and the retinoic acid receptor alpha (RARα) (de The et al., 1991; Goddard et al., 1991; Kakizuka et al., 1991; Pandolfi et al., 1991). Since its discovery in the early 1990s, PML has attracted intensive research attentions. A variety of physiological and pathological processes has been revealed related with PML cellular functions (Geoffroy and Chelbi-Alix, 2011; Carracedo et al., 2012; Chen et al., 2012; Ito et al., 2012).

**Figure 1:**
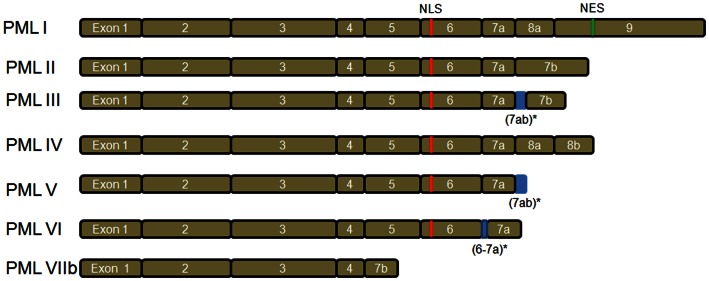
**PML isoforms**. There are seven main PML isoforms due to mRNA alternative splicing. PML III, PML V, and PML VI have retained intronic sequences (indicated by *). PML I to PML VI contain nuclear localization signal (NLS). The cytoplasmic PML VIIb is devoid of NLS. Notably, PML I contains both NLS and nuclear export sequence (NES).

There are seven main PML isoforms (PML I–PML VIIb) as described by Jensen et al. (2001) (Figure 1). PML I to PML VI contain the nuclear localization signal (NLS), resulting in the PML nuclear localization. The PML VIIb is missing the NLS and is likely localized in the cytoplasm. Notably, PML I also contains the nuclear export sequence (NES), which may lead to PML I localization in both nuclear and cytoplasm (Figure 1). Nuclear PML (nPML) is the central component of the sub-nuclear structure named PML nuclear bodies (PML-NBs) and it is essential for the formation and stability of PML-NBs, as loss of PML delocalizes the components of PML-NBs (Zhong et al., 1999, 2000). nPML regulates a variety of cellular functions including apoptosis (Guo et al., 2000; Lin et al., 2006), senescence (Pearson et al., 2000; Vernier et al., 2011; Martin et al., 2012), neoangiogenesis (Bernardi et al., 2006), DNA damage response (Dellaire and Bazett-Jones, 2004; Dellaire et al., 2006), and hematopoietic stem cell (HSC) maintenance (Ito et al., 2012). Despite the important role of PML in the nucleus, PML displays both nuclear and cytoplasm localization (Giorgi et al., 2010; Carracedo et al., 2011). In addition, the cytoplasmic PML (cPML) splicing isoforms devoid of NLS have been described in the cytoplasm (Jensen et al., 2001; Lin et al., 2004; Bernardi and Pandolfi, 2007; McNally et al., 2008). The cytoplasmic localization suggests PML may perform specific functions in the cytoplasm. As expected, the cellular functions of cPML are recently emerging and receiving great research attraction (Lin et al., 2004; McNally et al., 2008; Giorgi et al., 2010).

## cPML Regulates TGF-β Signaling Activation

An unexpected study by Lin et al. (2004) demonstrated the pivotal role of cPML in the activation of transforming growth factor β (TGF-β) signaling. The TGF-β is a well-known tumor suppressive factor, which induces the transcription of two cyclin inhibitors p15 and p21 and consequently causes cell growth arrest. In addition, TGF-β stimulation can induce apoptosis and cellular senescence (Katakura et al., 1999; Derynck et al., 2001; Siegel and Massague, 2003). Pioneering studies revealed that PML is also involved in cell proliferation, apoptosis, and senescence. Overexpression of PML inhibits cell growth *in vitro*, while *Pml*^−/−^ cells grow faster than wild-type counterparts (Mu et al., 1994; Wang et al., 1998a). On the other hand, *Pml*^−/−^ cells are resistant to multiple apoptotic stimulations (Wang et al., 1998b). PML is also required for oncogene induced premature aging (Ferbeyre et al., 2000; Pearson et al., 2000). The similarity of cellular functions indicates that PML and TGF-β signaling might be functionally interacted. Indeed, the TGF-β responses are dramatically impaired in *Pml*^−/−^ MEF cells (Lin et al., 2004). Surprisingly, the defects in TGF-β responses upon PML deficiency are rescued by the restoration of a cPML isoform (PML3 3–7), but not by a nuclear isoform PML4 (Lin et al., 2004). Although it is of note that nPML isoforms play a critical role in inhibiting cell growth and inducing apoptosis and senescence, the unexpected finding by Lin et al., suggests the important role of cPML in the modulation of TGF-β signaling and its cellular functions. Interestingly, TGF-β stimulation induces the expression of cPML in the cytoplasm, strengthening their cooperation in the cellular function regulation (Lin et al., 2004). Further investigation has demonstrated that cPML is required for the TβRI/TβRII/SARA/Smad complex assembly and its localization to the early endosome (Lin et al., 2004). Interestingly, nuclear sequestration of cPML by TG-interacting factor (TGIF) and c-Jun dissociates the complex formation and negatively regulates TGF-β signaling (Seo et al., 2006), indicating that the cytoplasmic localization is essential for cPML to activate TGF-β signaling. Consistent with this notion, relocation of cPML from nucleus to cytoplasm by PCTA attenuates the inhibitory effect of TGIF on the complex formation and TGF-β signaling (Faresse et al., 2008). Moreover, two nPML mutants devoid of their NLS localized in the cytoplasm fully rescue TGF-β transcriptional activity similar to the cPML isoform, further demonstrating the essential role of cytoplasmic localization for PML to regulate TGF-β signaling (Lin et al., 2004). Altogether, these studies demonstrate that PML cytoplasmic localization is critical for TGF-β signaling. Further efforts are required to reveal whether this phenomenon is universal for other cPML isoforms and the nucleus-cytoplasm relocated PML isoforms.

## cPML Regulates ER Calcium Release and Apoptosis Responses

Promyelocytic leukemia protein is required for multiple apoptosis pathways (Wang et al., 1998b). PML associated apoptosis has been primarily attributed to nPML or PML-NBs (Guo et al., 2000; Lin et al., 2006). PML interacts with tumor suppressor p53, recruits p53 to PML-NBs and potentiates p53-mediated gene expression and apoptosis (Guo et al., 2000). Two recent studies revealed that PML-mediated apoptosis also occurs outside the nucleus. The first study showed that cPML can be found in the early endosome, facilitates the assembly of the TβRI/TβRII/SARA/Smad2/3 complex and is critical for TGF-β-mediated apoptosis (Lin et al., 2004). Recently, another study by Giorgi et al. (2010) reported the enrichment of cPML at the endoplasmic reticulum (ER) and the mitochondria-associated membranes (MAMs), where are the ER and mitochondria contact sites, in primary MEFs. The MAMs are specialized microdomains important for the Ca^2+^ transfer between ER and mitochondria (Pinton et al., 2008). The Ca^2+^ transfer from ER to mitochondria is critical for ER stress induced cell fate determinant. During the early phase of ER stress, Ca^2+^ transfer may trigger an adaptive response by promoting mitochondria metabolism (Bravo et al., 2011), while Ca^2+^ overload in mitochondria leads to apoptotic cell death (Chami et al., 2008). Enrichment of cPML at MAMs microdomains facilitates the Ca^2+^ release from ER and consequently mediates apoptosis responses upon various stimuli, such as ER stress (Giorgi et al., 2010). In line with the PML-NBs in the nucleus and the cPML/TβRI/TβRII/SARA/Smad complex in the early endosome, cPML also forms a large complex containing PP2A/AKT/IP3R at MAMs to modulate the ER calcium release and apoptosis (Giorgi et al., 2010). However, it is unclear about which isoform of PML is enriched in the MAMs and facilitates apoptosis response. Further examination is needed to investigate whether nucleus-cytoplasm exported PML or bona fide cPML isoforms are involved in ER calcium release and apoptosis.

## cPML in Tumorigenesis: Tumor Suppressive or Oncogenic?

Several lines of evidence indicate that PML is a potential tumor suppressor. First, PML induces cell growth arrest, senescence, and apoptosis *in vitro* and inhibits oncogenic transformation *in vivo*. Second, PML-RARα fusion protein, an oncoprotein associated with the occurrence of most APL cases, serves as a dominant negative mutant to antagonize PML functions (Mu et al., 1994). The most important evidence that attests the role of PML as a tumor suppressor comes from the analysis of various genetic mouse models and human cancer samples. By using *Pml* deficiency mice as models, two studies showed that *Pml* deficiency promotes chemical-induced papilloma and lymphomas development as well as the lung cancer progression in RAS transgenic lung tumor model (Wang et al., 1998a; Scaglioni et al., 2006). Moreover, loss of *Pml* also synergizes with *Pten* inactivation to induce invasive prostate cancer development (Trotman et al., 2006). Finally, analysis of cancer specimens revealed that loss of PML protein expression is frequently detected in numerous human cancers (Gurrieri et al., 2004).

As mentioned above, cPML is essential for activation of the tumor suppressive TGF-β signaling and consequently inhibits cell growth, facilitates apoptosis and cell senescence (Lin et al., 2004). Consistently, MAMs cPML also promotes apoptosis through facilitating the ER calcium release (Giorgi et al., 2010). Both studies imply that cPML may also be a tumor suppressor similar to nPML does. Interestingly, the APL oncoprotein PML-RARα is expressed in both nuclear and cytoplasm (Kastner et al., 1992). The cPML-RARα disrupts cPML-Smad2/3 interaction and antagonizes the tumor suppressive TGF-β signaling, providing an additional mechanism for PML-RARα oncogenic function (Lin et al., 2004). It would be interesting to know whether PML-RARα would also antagonize MAMs cPML functions, thereby contributing to APL disease. Altogether, cPML likely serves as a tumor suppressor.

However, several reports on cPML mutants revealed their oncogenic potential. The PML truncated mutant was identified in the recurrent plasmcytoma cell cytoplasm and displayed oncogenic role, which may be due to a dominant negative effect (Zheng et al., 1998). More recently, two different PML mutations (1272delAG and IVS3–1G-A) have been identified in aggressive APL patients. Both mutations cause premature transcription stop before the NLS domain, thereby leading to the generation of cPML mutants (Gurrieri, 2004). Interestingly, these cPML mutants interact with and stabilize PML-RARα cytoplasmic complex, resulting in potentiating PML-RARα oncogenic function (Bellodi, 2006). Moreover, the two cPML mutants can induce the relocation of nPML to cytoplasm and inhibits p53 tumor suppressive ability (Bellodi et al., 2006). Altogether, these studies suggest that cPML may also be oncogenic. Consistent with this notion, several studies showed that cPML is upregulated in hepatocellular carcinoma (Terris et al., 1995; Chan et al., 1998), although it is unclear whether the cPML is derived from PML mutants, nPML relocation, or bona fide cPML isoforms. Therefore, in addition to the cPML mutants, further studies are needed to test whether the nucleus-cytoplasm relocated PML and bona fide cPML isoforms bear the similar oncogenic roles.

## The Role of cPML in Metabolism

Deregulated energy metabolism is a hallmark of human cancers. Aerobic glycolysis known as Warburg effect is highly utilized in cancers and shown to be an important driving force for cancer progression. The M2 isoform of pyruvate kinase (PKM2) is a glycolytic enzyme that catalyzes the dephosphorylation of phosphoenolpyruvate (PEP) to produce pyruvate, which will be converted into lactate rapidly. PKM2 is critical for aerobic glycolysis and expressed in proliferating cells during embryogenesis and tumorigenesis [reviewed in (Mazurek, 2011; Chaneton and Gottlieb, 2012)]. A recent study by Shimada et al. (2008) revealed that cPML may be involved in glycolysis through PKM2. cPML interacts with PKM2 in the cytoplasm, and the PML-2KA mutant that contains NLS mutations and localizes in the cytoplasm inhibits PKM2 activity and reduces lactate production (Shimada et al., 2008). Although it is unknown about which cPML isoform indeed interacts with PKM2 and regulates its activity, the work may provide a potential crosstalk between cPML and PKM2 in glycolysis regulation. It will be interesting to investigate whether cPML may participate in tumorigenesis through regulating PKM2 activity and glycolysis.

Two recent reports revealed that PML regulates fatty acid oxidation (FAO) (Carracedo et al., 2012; Ito et al., 2012). Pioneer studies demonstrated that FAO promotes ATP generation and cancer cell survival under metabolic stress, thus contributing to the tumor growth and survival (Schafer et al., 2009; Zaugg et al., 2011). Interestingly, loss of PML is correlated with the impairment of FAO and ATP production (Carracedo et al., 2012; Ito et al., 2012), and its overexpression promotes FAO, ATP generation, and cell survival in breast cells (Carracedo et al., 2012). Collectively, these studies suggest an unexpected survival role of PML through FAO regulation. It would be interesting to characterize which PML isoform regulates FAO and performs an unexpected tumor oncogenic role. Further studies are needed to examine whether cPML or nPML is involved in FAO and ATP generation.

## cPML Facilitates Antiviral Responses

Interferons (IFNs) play an important role in the antiviral responses. Upon viral infection, the cells release IFNs, which then bind to the cell surface specific receptors and activate downstream signaling to combat virus infection [reviewed in (Platanias, 2005)]. Interestingly, IFN treatment induces the expression of several components of PML-NBs including SP100, NDP52, and PML (Chelbi-Alix et al., 1995; Lavau et al., 1995) leading to increased number and size of PML-NBs. Notably, viral infection is prone to disrupt PML-NBs and delocalizes the components of PML-NBs (Puvion-Dutilleul et al., 1995a,b; Doucas et al., 1996; Korioth et al., 1996; Ahn and Hayward, 1997, 2000; Bell et al., 2000a,b). Further studies showed that the immediate-early protein (ICPo) of the herpes simplex virus type 1 (HSV-1) is localized to the PML-NBs and causes PML degradation leading to the disruption of PML-NBs (Everett et al., 1998; McNally et al., 2008). These studies imply that PML and PML-NBs may be involved in antiviral response. The supporting evidence came from a provoking study by Maroui et al. (2011). Maroui et al., found that PML IV interacts with 3D polymerase of encephalomyocarditis virus (EMCV) and recruits it to PML-NBs, thus inhibiting EMCV production. Surprisingly, EMCV resistance is specifically restricted to PML IV isoform, but not other PML isoforms (Maroui et al., 2011). However, it is unclear whether this PML isoform specificity also applies to other types of viral infection.

Emerging evidence suggests that cPML is also involved in the cellular resistance to viral infection. Studies conducted by different groups demonstrated that PML cytoplasmic relocation can be induced by the respiratory syncytial virus (RSV), the lymphocytic choriomeningitis virus (LCMV) and the human immunodeficiency virus type 1 (HIV-1) (Borden et al., 1998; Turelli et al., 2001; Brasier et al., 2004). cPML cooperates with the LCMV Z protein to inhibit eIF4E, which may reduce viral protein translation (Borden et al., 1998; Kentsis et al., 2001). Another study by Turelli et al. (2001) showed that cPML is implicated in HIV-1 transduction. HIV-1 infection redistributes PML together with the integrase interactor 1 (INI-1) from nucleus to cytoplasm, where PML and IN1-1 colocalize with HIV-1 preintegration complex and interfere with HIV-mediated transduction. The nucleus-cytoplasm redistribution of PML and INI-1 is dependent on the exportin (also known as Crm-1) transporting pathway. Interestingly, Crm-1 inhibitor leptomycin B blocks the nucleus-cytoplasm export of PML and INI-1 and increases the HIV-1 transduction (Turelli et al., 2001). Altogether, these reports demonstrate a crucial role of PML cytoplasmic relocation in the antiviral responses.

As the nucleus-cytoplasm transported PML plays crucial roles in the antiviral responses, it is not surprising that bona fide cPML isoforms may also display antiviral activity. Indeed, a recently study by McNally identified a novel bona fide cPML isoform (PML Ib) in HSV-1 infected cells (McNally et al., 2008). PML Ib sequesters the ICPo, which is involved in the viral transcription, in the cytoplasm, thereby repressing the viral replication. As a result, PML Ib overexpression greatly enhances the cellular resistance to HSV-1 infection. However, the predominantly nPML I isoform does not display antiviral ability against HSV-1. Further study showed that the loss of PML in *Pml*^−/−^ MEFs greatly enhances HSV-1 production compared with its wild type counterparts. Remarkably, restoration of PML Ib in *Pml*^−/−^ MEFs completely rescued this phenotype, indicating the PML Ib is sufficient for PML antiviral responses against HSV-1. The elegant study by McNally et al., reveals a critical role of cPML Ib in antiviral responses. It remains to be determined whether other cPML isoforms also perform dramatic viral defense ability against HSV-1, such as the cPML isoform involved in TGF-β signaling (Lin et al., 2004).

## cPML with Elusive Cytoplasmic Functions

In addition to the aforementioned cPML, there is another portion of cPML whose cytoplasmic functions still remain elusive. The most interesting finding is that the PML-NBs is dynamically regulated during cell cycle progress (Dellaire, 2006). PML localizes to the cytoplasm in mitosis phase and forms a specific complex termed mitotic accumulation of PML proteins (MAPPs) and then reforms PML-NBs in G1 phase. Typically, MAPPs is regarded as the transient depot of PML-NBs dynamic regulation through cell cycle progression (Dellaire, 2006). It will be interesting to identify whether the MAPPs may have specific cytoplasmic functions.

The redistribution of nPML to cytoplasm has also been described in the laminopathy diseases characterized by *LMNA* gene mutations. PML cytoplasmic particles (PML-CPs) is found in the laminopathy cells and the number of these particles increases with disease severity (Houben et al., 2013). The PML-CPs may come from the PML-NBs redistribution, since laminopathies could lead to nuclear rupture. However, in the laminopathy cells without overt abnormal nucleus, the number of PML-CPs are also significantly increased (Houben et al., 2013), suggesting that PML-CPs may also from in the cytoplasm. In this regard, it is still unknown whether bona fide cPML isoforms are involved in the formation of PML-CPs. Further research is needed to identify the origin of PML in the PML-CPs and to investigate whether PML-CPs have direct effect to promote laminopathies.

## Conclusion and Perspectives

**Figure 2:**
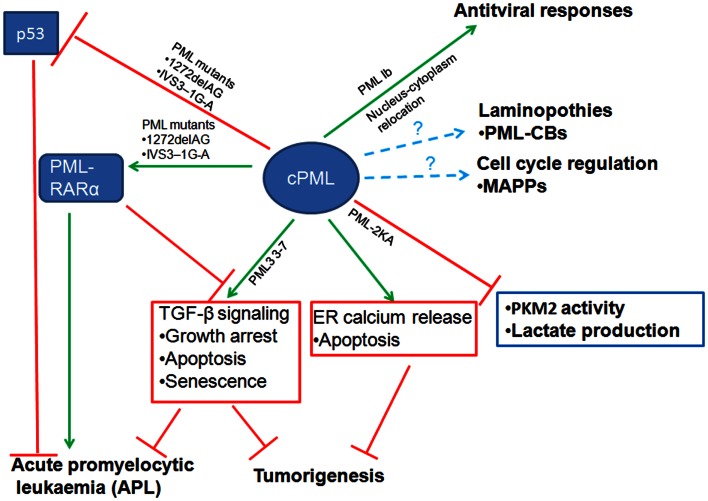
**Diverse cellular functions of cytoplasmic PML (cPML)**. cPML displays both tumor suppressive and oncogenic functions. On one hand, cPML increases TGF-β signaling or ER Ca^2+^ release to suppress tumorigenesis. On the other hand, cPML inhibits tumor suppressor p53 or potentiates oncoprotein PML-RARα to promote tumorigenesis in acute promyelocytic leukemia (APL). cPML may also regulate glycolysis and tumorigenesis through the inhibition of PKM2 activity and lactate production. In addition, cPML is involved in antiviral responses, laminopothies, and cell cycle regulation.

The emerging evidence we discussed in this review revealed that cPML exhibits multiple cellular functions (Figure 2). The cPML can be derived from nucleus-cytoplasm redistribution, bona fide cPML isoforms or nPML mutations which lose nuclear import ability. It is eager for the field to know whether the cellular functions regulated by cPML are related to specific cPML isoforms or nucleus-cytoplasm redistribution PML. In addition, to further address the important role of cPML *in vivo*, cPML specific animal models such as cPML knockin mice or nPML knockin mice are needed. Another remaining question is how cPML is regulated to orchestrate downstream events. Given that PML undergoes distinct posttranslational modifications [reviewed in (Carracedo et al., 2011)], it is very likely that cPML may require certain posttranslational modifications to perform proper cellular functions.

## Conflict of Interest Statement

The authors declare that the research was conducted in the absence of any commercial or financial relationships that could be construed as a potential conflict of interest.
